# Metallic Bond Induces Soft Phonon Mode and Anharmonicity in Heusler Alloy

**DOI:** 10.1002/advs.202509238

**Published:** 2025-07-14

**Authors:** Hao‐Xuan Liu, Hai‐Le Yan, Nan Jia, Bo Yang, Zongbin Li, Xiang Zhao, Liang Zuo

**Affiliations:** ^1^ Key Laboratory for Anisotropy and Texture of Materials (Ministry of Education) School of Material Science and Engineering Northeastern University Shenyang 110819 China

**Keywords:** anharmonicity, long‐range interaction, metallic bonding, metastable Heusler alloys, soft mode

## Abstract

The fruitful functional performances of metastable Heusler alloys originate from the martensitic transformation driven by the transverse acoustic soft phonon mode, regulated by anharmonicity. However, the origins of the soft mode and the anharmonicity in these alloys remain unclear. In this work, using *ab‐initio* calculations combined with self‐consistent phonon theory (SCPH) and compressed sensing techniques in machine learning, a link among chemical bonding, long‐range interaction, soft mode, and anharmonicity is established using Ni_2_MnGa as a model system. The long‐range interaction rooted in metallic bonding between spin‐down *d*‐orbitals of Ni─Ni induces phonon softening and significant anharmonicity. This finding offers new insights into the role of metallic bonding and long‐range interactions in martensitic transformation, providing a fresh perspective for the design and optimization of phase transition functional alloys.

## Graphical abstract

The fruitful functional performances of metastable Heusler alloys originate from the martensitic transformation driven by the transverse acoustic soft phonon mode, regulated by anharmonicity. This study reveals that the long‐range interaction rooted in metallic bonding between spin‐down *d*‐orbitals of Ni─Ni induces phonon softening and significant anharmonicity, which provides a fresh perspective for the design and optimization of phase transition functional alloys.

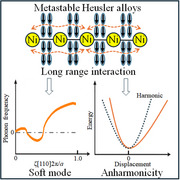

## 1 Introduction

Metastable Heusler alloys represented by Ni_2_MnGa exhibit various excellent functional properties such as magnetic shape memory effect, elastocaloric effect, and superelasticity, making them attractive for applications in intelligent sensors, solid‐state cooling, and aerospace.^[^ ^1^, ^2^, ^3^, ^4^ ^]^ Central to these extraordinary properties is the martensitic transformation, which is driven by phonon soft modes.^[^ ^5^, ^6^ ^]^ Consequently, investigating lattice vibrations is crucial for understanding the phase transition mechanism.

In Ni_2_MnGa, numerous studies^[^ ^5^, ^6^, ^7^, ^8^, ^9^, ^10^ ^]^ have demonstrated that the pre‐martensitic phase transition ≈260 K is induced by the softening of the transverse acoustic (TA_2_) mode at [1/3,1/3,0] (2π/*a*) polarized along [110]. This phenomenon has been extensively observed using various experimental techniques, including neutron scattering,^[^ ^5^, ^6^ ^]^ X‐ray diffraction,^[^ ^7^ ^]^ electron microscopy,^[^ ^8^ ^]^ and UV‐photoemission measurements.^[^ ^9^ ^]^ Computational studies, such as those by Zayak et al.,^[^ ^10^ ^]^ successfully reproduced this phonon dispersion through *ab‐initio* calculations. The origin of these soft modes has been widely attributed to the nested Fermi surfaces.^[^ ^9^, ^11^, ^12^, ^13^, ^14^, ^15^ ^]^ However, a notable discrepancy exists: the observed nesting vector (≈0.40) does not match the reported soft mode at *q* = 0.33 in Ni_2_MnGa.^[^ ^14^, ^15^ ^]^ Similar soft modes have also been observed in Ni‐Mn‐In and Ni‐Mn‐Sn et al.^[^ ^16^, ^17^ ^]^ In contrast, Co‐based Heusler alloys, such as Co_2_MnGa,^[^ ^16^ ^]^ do not exhibit soft modes and thus the associated functional behaviors, highlighting a fundamental difference between the Ni‐ and Co‐based systems. However, the underlying reason for this difference remains unclear.

In addition to soft mode, metastable Heusler alloys exhibit significant anharmonicity, which coexists with the soft mode. As the temperature decreases, the TA_2_ acoustic mode softens, while it hardens upon heating.^[^ ^5^, ^6^ ^]^ This temperature‐driven behavior suggests that anharmonicity plays a key role in tailoring the soft mode. It is known that the anharmonicity significantly affects thermal conductivity,^[^ ^18^, ^19^, ^20^ ^]^ thermal expansion,^[^ ^21^, ^22^ ^]^ and other functional properties^[^ ^23^, ^24^ ^]^ in thermoelectric,^[^ ^25^, ^26^ ^]^ superionic^[^ ^27^, ^28^ ^]^ and ferroelectric^[^ ^24^, ^29^ ^]^ materials. However, the knowledge about the role of anharmonicity on phonon hardening and martensitic transformation remains limited. Experimentally, distinguishing between harmonic and anharmonic contributions is challenging, while computational studies have primarily focused on harmonic and quasiharmonic (QHA) approximations. A deeper understanding of both soft mode and anharmonicity is crucial for the systematic design and optimization of functional Heusler alloys.

In this work, we explore the origin of soft modes and anharmonicity of metastable Heusler alloys using *ab‐initio* calculations combined with self‐consistent phonon theory (SCPH)^[^ ^30^ ^]^ and compressed sensing techniques.^[^ ^31^ ^]^ These advanced methods are particularly effective for studying anharmonicity and soft modes and have recently led to several significant works.^[^ ^32^, ^33^, ^34^ ^]^ A comparative study was conducted on X_2_MnGa (X = Ni, Co) alloys as representatives with/without phase transformation. Our results reveal that the softening of the transverse acoustic mode and the associated anharmonicity originate from long‐range Ni─Ni interactions. These interactions are primarily driven by metallic bonding with a strong contribution from spin‐down *d*‐states.

## 2 Methods

All density functional theory (DFT) calculations were performed using *Vienna ab initio Simulation Package (VASP)* ^[^ ^35^ ^]^ within the projector augmented wave (PAW) approach.^[^ ^36^ ^]^ The Perdew‐Burke‐Ernzerhof (PBE) generalized gradient approximation (GGA)^[^ ^37^ ^]^ functional was used with a plane wave cutoff of 600 eV. The energy and force convergence criteria were set to 10^−8^ eV and −10^−7^ eV Å^−1^, respectively. Gamma centered *k*‐spacing value of 0.02 × 2π Å^−1^ was used for the phonon calculations. Harmonic interatomic force constants (IFCs) were obtained using the finite‐displacement approach by ALAMODE package,^[^ ^38^ ^]^ with a displacement of 0.01 Å in a 5 × 2 × 2 supercell (Figure S1, Supporting Information) constructed from a tetragonal crystallographic unit cell.^[^ ^10^ ^]^ For anharmonic IFCs we first sample 80 snapshots from a 4000‐step *ab‐initio* molecular dynamics (AIMD) simulation at 300 K with a time step of 2 fs in the same 5 × 2 × 2 supercell. The same *k*‐point spacing as that used in the phonon calculations was applied. To ensure access to the anharmonic regime, an additional atomic displacement of 0.1 Å in random directions was applied to each sampled AIMD snapshot to generate quasirandom configurations. The Hellmann–Feynman forces for these configurations were then computed using static DFT. Finally, the anharmonic IFCs were estimated from the displacement‐force dataset of 80 quasirandom configurations using the compressive sensing (CS) technique,^[^ ^31^ ^]^ using the least absolute shrinkage and selection operator (LASSO) regression method.^[^ ^39^ ^]^ Through four‐fold cross‐validation, the optimal regularization parameter was determined to be α = 8.98089 × 10^−7^, yielding a cross‐validation fitting error of less than 2%. Finite‐temperature phonon dispersions were computed using the self‐consistent phonon theory (SCPH).^[^ ^30^ ^]^ The Bubble correction is on top of the SCPH dynamical matrix.^[^ ^30^ ^]^ The phonon dispersions were subsequently recalculated after modifying the force constants using Phonopy.^[^ ^40^, ^41^ ^]^

## 3 Results and Discussion

### 3.1 Soft Phonon Mode and Anharmonicity

**Figure 1:**
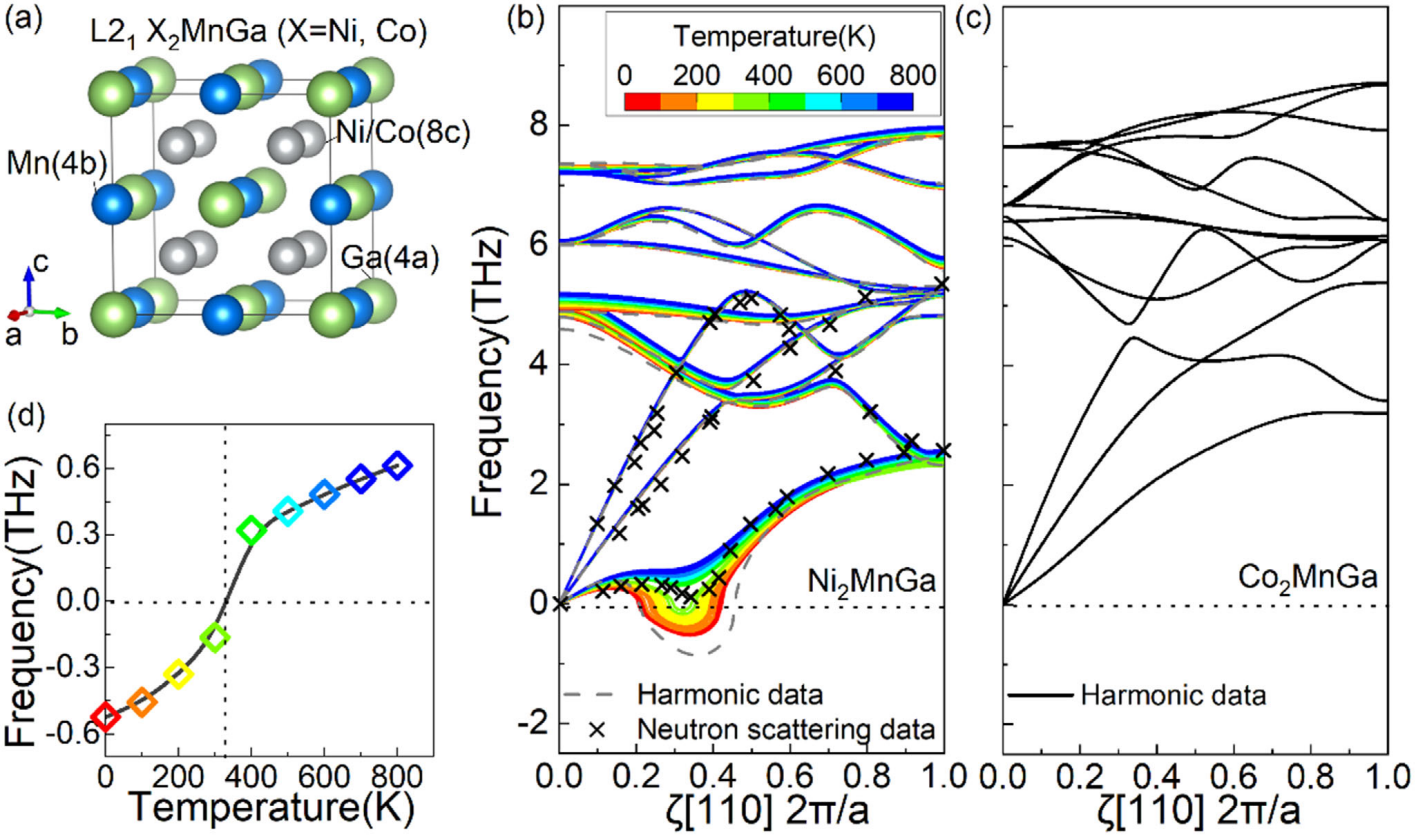
Crystal structure and phonon dispersion of X_2_MnGa (X = Ni, Co). a) L2_1_ crystal structure. Phonon dispersion relations for b) Ni_2_MnGa, showing harmonic data (black dashed lines), finite temperature data from SCPH (colorful solid lines), and neutron data (gray crosses) from Ref.,[^[^ ^5^ ^]^] and for c) Co_2_MnGa, showing harmonic data. d) Temperature dependence of the TA_2_ soft‐mode frequency at [1/3,1/3,0] (2π/*a*) in Ni_2_MnGa.

Heusler‐type X_2_MnGa (X = Ni, Co) alloys crystallize in cubic L2_1_ structure in austenitic with four face‐centered‐cubic (FCC) sublattices: Ga at *4a* (0 0 0), Mn at *4b* (0.5 0.5 0.5), and X at *8c* (0.25 0.25 0.25) and (0.75 0.75 0.75), as shown in **Figure** **1**a. The harmonic phonon dispersion curves along [110] for Ni_2_MnGa (gray dashed line) and Co_2_MnGa (black solid line) are presented in Figure 1b,c, respectively. While the phonon curves of the two alloys are highly similar, Ni_2_MnGa exhibits a soft mode in the TA_2_ branch, whereas Co_2_MnGa does not, which is consistent with the results of previous work.^[^ ^5^, ^6^, ^16^, ^17^ ^]^ By the SCPH method, the finite‐temperature phonon dispersion curves (color lines in Figure 1b) of Ni_2_MnGa are calculated. Notably, the frequency of TA_2_ soft mode exhibits significant temperature dependence. The phonon dispersion at 300K is consistent with the neutron scattering data (black cross in Figure 1b) reported in Ref.[^[^ ^5^ ^]^]

Figure 1d shows the variation of TA_2_ soft mode frequency of Ni_2_MnGa at [1/3,1/3,0] (2π/*a*) with temperature. Notably, the frequency gradually increases with rising temperature, highlighting a strong anharmonicity in Ni_2_MnGa. The soft‐mode frequency reaches zero ≈300 K, which is consistent with the experimentally observed pre‐martensitic transition temperature (≈260 K).^[^ ^5^, ^6^, ^9^ ^]^ The square of the soft‐mode frequency is found to be linearly related to temperature (Figure S2, Supporting Information), which is consistent with previous neutron scattering data.^[^ ^5^ ^]^

### 3.2 Anharmonicity from Ni─Ni

**Figure 2:**
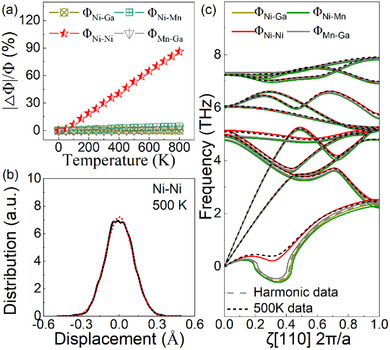
Anharmonicity and its impact on soft mode. a) Variation ratio of projected IFC of Ni─Ga, Ni─Mn, Ni─Ni and Mn─Ga bonds (Φ_Ni‐Ga_, Φ_Ni‐Mn_, Φ_Ni‐Ni_ and Φ_Mn‐Ga_) with respect to temperature. b) Bond length distribution of adjacent Ni─Ni pairs (solid line) at 500 K extracted from AIMD trajectories at each time step. The dotted lines are fitted with a Gaussian relation. Their deviation represents the anharmonicity.^[^ ^42^ ^]^ c) Recalculated phonon dispersion after replacing harmonic IFCs by the effective IFCs at 500K for different atom pairs. The on‐site force constants were adjusted to satisfy the acoustic sum rule (ASR).^[^ ^38^ ^]^

To unravel the impact of anharmonicity on IFCs, the SCPH phonon dispersions were fitted to obtain effective IFCs that include the renormalization for anharmonicity. The projected IFCs were further obtained by calculating the projection of the effective (renormalized) IFCs matrix along the direction of the position vector between the atom pair (see Note S1, Supporting Information for details). **Figure** **2**a shows the temperature‐dependent variation of projected IFCs of Ni─Ga, Ni─Mn, Ni─Ni and Mn─Ga bonds (Φ_Ni‐Ga_, Φ_Ni‐Mn_, Φ_Ni‐Ni_ and Φ_Mn‐Ga_). Among these, Φ_Ni‐Ni_ exhibits the most significant changes with increasing temperature, while the variations in Φ_Ni‐Ga_, Φ_Ni‐Mn_ and Φ_Mn‐Ga_ are limited. This suggests that the anharmonicity in Ni_2_MnGa might appear to be primarily associated with the Ni─Ni pair.

Figure 2b shows the distributions of Ni─Ni bond lengths extracted from the AIMD trajectories at 500K (see Note S2, Supporting Information for details). Clearly, the distributions of Ni─Ni bond length exhibit observable deviations from Gaussian behavior (dotted line), indicating the presence of non‐negligible anharmonic interactions. In contrast, the bond length distributions of Ni─Ga, Ni─Mn, and Mn─Ga almost coincide with harmonicity‐associated Gaussian distributions (see Figure S3, Supporting Information). These results, aligning well with the analysis of effective IFCs, evidence that the anharmonicity originates from Ni─Ni interactions.

To clarify the impact of anharmonic interactions on the soft phonon mode, the phonon dispersions were recalculated by replacing the harmonic IFCs of Ni─Ga, Ni─Mn, Ni─Ni, and Mn─Ga with their corresponding anharmonicity‐contained effective IFCs at 500 K. During the calculations, rather than only updating the nearest‐neighbor interactions, the IFCs of atom pairs at all distances were replaced. As shown in Figure 2c, introducing the effective IFCs of Ni─Ga, Ni─Mn, and Mn─Ga resulted in only minor changes in the soft mode compared to its harmonic counterpart. In contrast, substituting the effective IFCs of Ni─Ni results in significant changes, bringing the soft phonon mode closer to that observed at 500 K, which confirms the anharmonicity origin of Ni─Ni interaction.

### 3.3 Long‐Range Interactions

**Figure 3:**
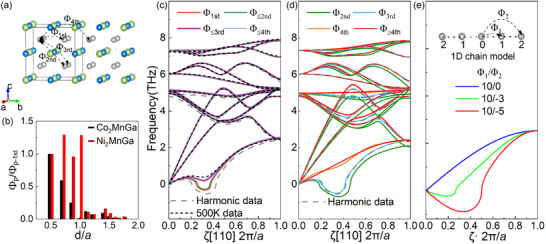
Impact of the long‐range interactions on the acoustic soft mode. a) Schematic diagram of IFCs of Ni─Ni (Co─Co) for the first four nearest neighbors in the crystal structure. b) Normalized harmonic IFCs with respect to the 1st IFCs vs. atomic distances for X_2_MnGa (X = Ni, Co). Larger values of the 2nd and 3rd IFCs against the nearest one in Ni_2_MnGa may be associated with long‐range electron‐mediated interactions (similar to the RKKY mechanism). c) Recalculated phonon dispersion by replacing the harmonic IFCs between Ni─Ni for the first *n* (*n* ∈{1, 2, 3, 4}) neighbors (Φ_1st_, Φ_≤2nd_, Φ_≤3rd_ and Φ_≤4th_) with their corresponding effective ones at 500 K. d) Recalculated phonon dispersion after setting different Ni─Ni long‐range interactions (Φ_2nd_, Φ_3rd_, Φ_4th_ and Φ_≥4th_) to zero, while adjusting the on‐site IFCs to satisfy the ASR. e) Phonon dispersion in a model 1D atomic chain, two numbers in the legend represent relative interaction strengths of the first (Φ_1_) and second neighbors (Φ_2_).

After identifying Ni─Ni interactions as the origin of anharmonicity in Ni_2_MnGa, we compared the impact of the IFCs of Ni─Ni in Ni_2_MnGa with that of IFCs of Co─Co in Co_2_MnGa. The lattice constants of Ni_2_MnGa and Co_2_MnGa are 5.80 and 5.71 Å, respectively. As shown in **Figure** **3**b, the projected IFCs of Ni─Ni in Ni_2_MnGa fluctuate as distance increases and do not show significant decay up to the fourth nearest neighbor (illustrated schematically in Figure 3a). Only beyond this range, noticeable decay occurs. In contrast, for the projected IFCs of Co─Co in Co_2_MnGa, it continuously decreases with increasing distance. These findings indicate that the Ni─Ni interaction in Ni_2_MnGa is a long‐range one, which is absent in Co_2_MnGa.

To quantify the anharmonic interactions of Ni─Ni at different distances, the phonon dispersion by replacing the harmonic IFCs of Ni─Ni for the first *n* (*n* ∈ {1, 2, 3, 4}) neighbors (Φ_1st_, Φ_≤2nd_, Φ_≤3rd_ and Φ_≤4th_) with their corresponding effective one at 500 K were calculated, respectively. In Figure 3c, replacing Φ_1st_ alone results in a slight increase in the frequency of the soft mode. Extending this replacement to Φ_≤2nd_ does not further affect the frequency. In contrast, substituting Φ_≤3rd_ leads to a significant increase in the frequency of soft mode, aligning closer to the soft mode frequency observed at 500 K. Replacing Φ_≤4th_ yields no further notable changes beyond Φ_≤3rd_. These findings suggest that apart from short‐range interaction Φ_1st_, the interactions of Φ_3rd_ play a critical role in hardening the soft mode. In other words, Φ_3rd_ tends to stabilize the austenite at high temperatures. Thus, Φ_3rd_ could take the dominant role in the reverse martensitic transformation from low‐temperature martensite to high‐temperature austenite. The significance of Φ_3rd_ may be attributed to its larger temperature‐dependent variation compared to Φ_2nd_ and Φ_4th_ (Figure S4a, Supporting Information).

In the main‐group IV–VI materials,^[^ ^43^ ^]^ such as SnTe and Bi_2_Te_3_, the long‐range interactions are known to drive optical soft‐mode behavior. To clarify the role of long‐range interactions of Ni─Ni in Ni_2_MnGa on the transverse acoustic (TA_2_) soft mode, the phonon dispersions were recalculated after setting different long‐range interactions (Φ_2nd_, Φ_3rd_, Φ_4th_ and Φ_≥4th_) to zero and adjusting the on‐site IFCs accordingly. From Figure 3d, we see minor changes in soft mode when Φ_2nd_ or Φ_3rd_ is removed. However, removing Φ_4th_ nearly eliminates the soft mode, and removing Φ_≥4th_ completely eliminates it. This indicates that Φ_4th_ is the primary source of the TA_2_ soft mode. Therefore, in contrast to Φ_3rd_, Φ_4th_ tends to destabilize the austenite and thus could be the engine for martensitic transformation from austenite to martensite. Further analysis reveals that the Φ_4th_ is negative, while Φ_1st_, Φ_2nd_ and Φ_3rd_ are positive (Figure S4b, Supporting Information). As explained in Note S1 (Supporting Information), positive IFCs generally stabilize the crystal structure, whereas negative IFCs (Φ_4th_) promote structural instability and are responsible for soft‐mode behavior. Notably, with increasing temperature, Φ_1st_ and Φ_3rd_ become more positive, which aligns with their stabilizing effect on the soft mode in Figure 3c.

To generalize our conclusion regarding the role of long‐range interactions in softening the TA mode, we employed a 1D lattice chain model (see Note S3, Supporting Information for details). In this model, the first‐ (Φ_1_) and second‐ (Φ_2_) nearest neighbor (Figure 3e) corresponded to the first‐ (Φ_1st_) and fourth‐ (Φ_4th_) interactions along the ⟨100⟩ direction in Ni_2_MnGa (Figure 3a), respectively. Figure 3e shows that when Φ_1_ remains constant and Φ_2_ increases, the TA phonon frequency decreases from positive to negative between the zone center and boundary, matching the soft‐mode behavior in Ni_2_MnGa. These results further confirm that long‐range interactions predominantly induce the TA soft mode, suggesting that this mechanism may extend beyond metastable Heusler alloys.

### 3.4 Electron Structure

**Figure 4:**
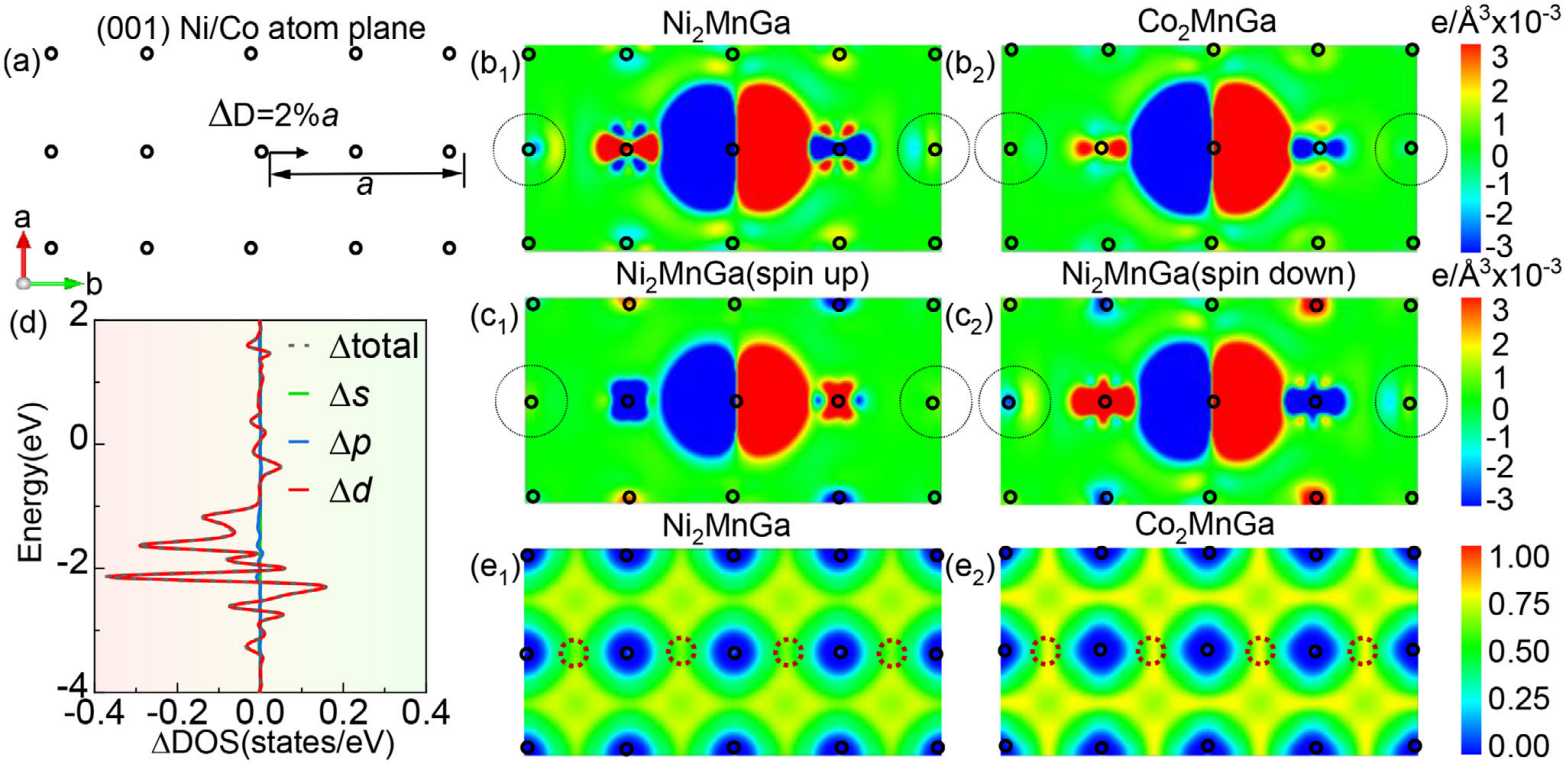
Electron structure of X_2_MnGa (X = Ni, Co). a) Schematic of the atom plane displacement along the (001) plane. b_1_,b_2_) Changes in charge density due to atom displacement in Ni_2_MnGa and Co_2_MnGa in (001) plane, respectively. c_1_,c_2_) Changes in spin up and spin down electron of Ni_2_MnGa in (001) plane, respectively. d) Density of states (DOS) change for spin‐down electrons for the fourth‐nearest neighbor Ni atom in Ni_2_MnGa due to displacement. e_1_,e_2_) Electron localization function (ELF) of Ni_2_MnGa and Co_2_MnGa in (001) plane, respectively.

To unravel the electron origin of the long‐range interaction, we calculated the changes in electron structure induced by atomic displacement in X_2_MnGa (X = Ni, Co). **Figure** **4**a illustrates a 2% displacement (along [010] direction) of the central atom in the (001) plane toward its fourth‐nearest neighbor. Figure 4b_1_,b_2_ compare the resulting changes in the charge density for Ni_2_MnGa and Co_2_MnGa. As shown in Figure 4b_1_, the Ni_2_MnGa exhibits significant electron polarization extending up to the fourth‐nearest neighbors (marked by dot black circles). In contrast, in Co_2_MnGa (Figure 4b_2_), the electron polarization is short‐ranged, with no obvious perturbation to the fourth‐nearest neighbors. These results confirm that the Ni─Ni interactions in Ni_2_MnGa exhibit long‐range behavior, contrasting with the short‐range nature of Co–Co interactions in Co_2_MnGa, consistent with Figure 2b. Further analysis shows that the long‐range Ni–Ni interaction in Ni₂MnGa induces a charge density perturbation of ≈2.0×10^−3^ e/Å^3^ at ≈5.8 Å, which is comparable to that of typical long‐range anharmonic interaction in thermoelectric materials, such as the Pb–Te interaction in PbTe (≈1.5 e/Å^3^×10^−3^ at ≈6.3 Å).^[^ ^43^ ^]^

To clarify the different spin state contributions to these long‐range interactions in Ni_2_MnGa, electron density distributions of spin up and spin down due to displacement were calculated. Figure 4c_1_,c_2_ show negligible changes in spin‐up electron density, whereas significant perturbations appear in spin‐down states at the fourth‐nearest neighbors, consistent with the changes in the total electron density. Their results suggest that the long‐range interactions in Ni_2_MnGa primarily originate from the spin‐down electrons.

Next, to explore the orbital origin of these long‐range interactions, we calculated the difference in density of states (DOS) for spin‐down electrons for the fourth‐nearest neighbor Ni atom in Ni_2_MnGa before and after displacement, as shown in Figure 4d. The analysis reveals that changes in *d* orbital DOS (Δ*d*) closely follow the total changes in DOS (Δtotal), while the contributions from *p* (Δ*p*) and *s* (Δ*s*) orbitals are negligible. This indicates that the long‐range interactions in Ni_2_MnGa originate from the Ni *d* orbitals, in contrast to the *p* orbital resonance bonds typical in main‐group compounds.^[^ ^43^ ^]^ The electron localization function (ELF) of X_2_MnGa (X = Ni, Co) was used to analyze the bonding characteristics, as shown in Figure 4e_1_,e_2_. We focus on the ELF of the X─X bond center position (highlighted by the red circle). The ELF of Ni─Ni is ≈0.5, indicating metallic bonding, while the ELF of Co─Co is ≈0.75, suggesting a more covalent bonding nature. The formation of metallic bonds through *d*‐*d* hybridization in Heusler alloys has also been associated with intrinsic ductility in previous studies,^[^ ^44^, ^45^, ^46^ ^]^ and is analogous to *d* orbital delocalization mechanisms observed in catalytic systems.^[^ ^47^, ^48^ ^]^ These results suggest that the long‐range interactions in Ni_2_MnGa arise from metallic bonding between spin‐down *d*‐orbitals of Ni─Ni. In the framework of solid‐state physics,^[^ ^49^ ^]^ such interactions can be understood through the concept of delocalized electrons in metals: valence electrons form a “sea” that allows indirect coupling between positively charged ion cores over extended distances.

## 4 Conclusion

In this study, we reveal the origin of soft modes and anharmonicity in metastable Heusler alloys. Our findings demonstrate that the soft modes and anharmonic behavior originate from long‐range Ni─Ni interactions. The results from 1Datomic chain models highlight that this mechanism may also apply to systems beyond metastable Heusler alloys. The electron structure analysis further reveals that the significant long‐range interactions in Ni_2_MnGa arise from metallic bonding, particularly from spin‐down *d* states. These insights offer a new perspective on the role of long‐range interactions and metallic bonding in the design and optimization of metastable Heusler alloys. Our work clarifies the origin of acoustic soft modes and deepens the understanding of lattice dynamics.

## Conflict of Interest

The authors declare no conflict of interest.

## Supporting information



Supporting Information

## Data Availability

The data that support the findings of this study are available from the corresponding author upon reasonable request.
