# Supplementary material for: Metallic Bond Induces Soft Phonon Mode and Anharmonicity in Heusler Alloy
Source: Adv Sci (Weinh). 2025 Jul 14;12(38):e09238. doi: 10.1002/advs.202509238 (PMC12520559; doi:10.1002/advs.202509238)
Supplement: Supplementary file 1 — Supporting Information [file ADVS-12-e09238-s001.pdf]

ADVANCED SCIENCE
Open Access

Supporting Information

for *Adv. Sci.*, DOI 10.1002/advs.202509238

Metallic Bond Induces Soft Phonon Mode and Anharmonicity in Heusler Alloy

Hao-Xuan Liu, Hai-Le Yan, Nan Jia, Bo Yang, Zongbin Li, Xiang Zhao and Liang Zuo*

Metallic bond induces soft mode and anharmonicity in metastable Heusler alloys

Supplementary materials

Hao-Xuan Liu, Hai-Le Yan,† Nan Jia, Bo Yang, Zongbin Li, Xiang Zhao, Liang Zuo

Key Laboratory for Anisotropy and Texture of Materials (Ministry of Education), School of Material Science and Engineering, Northeastern University, Shenyang 110819, China

Supplementary Figures

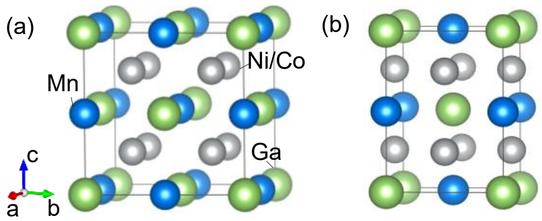

Fig. S1. Illustrations of structural models for the Ni_2MnGa alloys. (a) conventional cell, (b) tetragonal crystallographic unit cell.

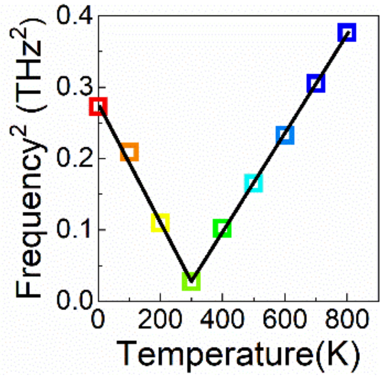

Fig. S2. Temperature dependence of the square of the soft-mode frequency at [1/3,1/3,0] ($2\pi/a_0$) in Ni_2MnGa. The square of the soft-mode frequency is found to be linearly related to temperature, which is consistent with previous neutron scattering data [1]

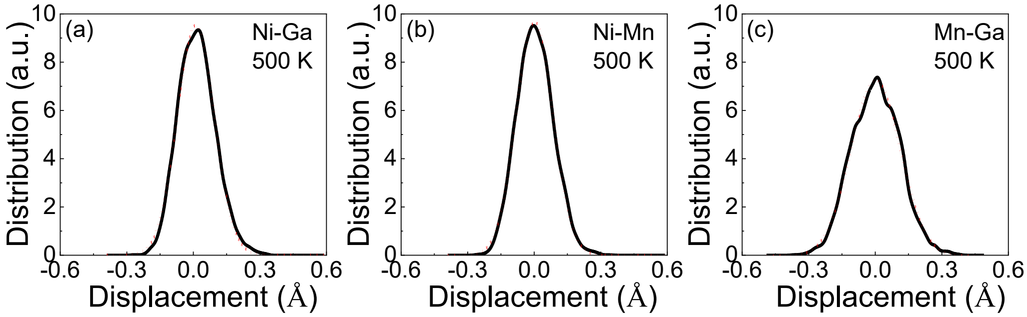

Fig. S3. Anharmonicity of Ni-Ga, Ni-Mn and Mn-Ga from AIMD. Distribution of bond length (solid line) corresponding to (a) Ni-Ga, (b) Ni-Mn and (c) Mn-Ga at 500K. These distributions were extracted from the same AIMD trajectories. At each time step, the distances corresponding to Ni-Ga, Ni-Mn Mn-Ga are computed, respectively. The dotted lines represent the best Gaussian fit to the calculated atomic distributions. The bond length distributions of Ni-Ga, Ni-Mn, and Mn-Ga almost coincide with Gaussian distributions, suggesting a harmonic interaction.

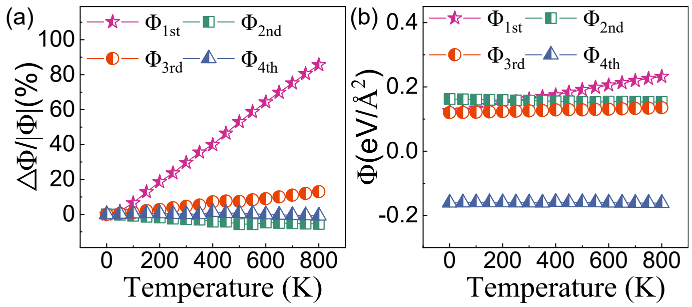

Fig. S4. Temperature dependence of the projected IFCs (contain sign) of Ni-Ni for the first four nearest neighbors. (a) $\triangle\Phi/|\Phi|$; The Φ_{1st} and Φ_{3rd} exhibit a larger change with increasing temperature compared to Φ_{2nd} and Φ_{4th}, which corresponds to the changes of soft-mode frequency in recalculated phonon desperation. (b) Φ. the Φ_{4th} is positive, whereas Φ_{1st}. Φ_{2nd} and Φ_{3rd} are negative. The sign of the Φ is defined in Note 1. A negative IFC tends to promote displacement expansion, contributing to structural instability (Φ_{4th}) whereas positive IFCs stabilize the crystal structure as explained (Φ_{1st}. Φ_{2nd} and Φ_{3rd}).

Supplementary Notes

Supplementary Note 1. Projected IFCs

The projected IFCs Φ_p are calculated from effective (renormalized) harmonic force constants, generated as Cartesian force constant matrices for each atom pair k, k'. As such, they can easily be projected along their respective interatomic vectors, as outlined in Ref.[2]:

$$\Phi_p(kk') = \left| \begin{pmatrix} \Phi_{kk'}^{xx} & \Phi_{kk'}^{xy} & \Phi_{kk'}^{xz} \\ \Phi_{kk'}^{yx} & \Phi_{kk'}^{yy} & \Phi_{kk'}^{yz} \\ \Phi_{kk'}^{zx} & \Phi_{kk'}^{zy} & \Phi_{kk'}^{zz} \end{pmatrix} \frac{r(k') - r(k)}{|r(k') - r(k)|} \right|$$

where $\Phi_{kk'}^{\alpha\beta}$ $(\alpha, \beta \in \{x, y, z,\})$ is defined as:

$$\Phi_{kk'}^{\alpha\beta} = \frac{\partial^2 V}{\partial r_k^\alpha \partial r_{k'}^\beta} = \frac{\partial F_{k'}^\beta}{\partial r_k^\alpha}$$

It can be analyzed that if the IFC is positive, the atom k' could experience a force in the same direction as the displacement of atom k, which tends to reduce relative displacement between atoms k and k', thereby stabilizing the crystal structure. In contrast, negative IFCs contribute to structural instability.

To further analysis the sign of the IFCs, the normalized trace of IFC (nIFC) [3] is introduced as follows:

$$nIFC = \frac{\Phi_{kk\prime}^{xx} + \Phi_{kk\prime}^{yy} + \Phi_{kk\prime}^{zz}}{\Phi_{kk}^{xx} + \Phi_{kk}^{yy} + \Phi_{kk}^{zz}}$$

In Fig. S4, the sign of the Φ's is defined as the same as sign of *n*IFC.

Supplementary Note 2. Bond lengths from AIMD

The bond lengths for Ni-Ga, Ni-Mn, Ni-Ni and Mn-Ga were extracted from the same AIMD trajectories. The time step of simulations is 1.0 fs. The system is first equilibrated at 500 K in an NVT ensemble for 5 ps. Then, it is put into an NVE ensemble at 100 ps to collect data on bond lengths.

Supplementary Note 3 Phonon dispersions of simple 1-dimensional chain

To demonstrate that the long-ranged interaction can induce the softening of the transverse acoustic (TA) mode, we calculated the phonon dispersion of a simple one-dimensional (1D) chain. Here, we provide a detailed description of the calculations.

The phonon frequency can be calculated by

$$\omega(q) = \sqrt{\frac{D(q)}{M}}$$

where M is the atomic mass (assumed as 1 for simplicity). The dynamical matrix $D(q)$ in the diatomic crystal is：

$$D(q)=2[\Phi_1(1-\cos(qa))+\Phi_2(1-\cos(2qa))]$$

where a is the lattice spacing, which is set to 1 for simplicity, q is the wavevector, which can take values in the first Brillouin zone $q\in[-\pi/a,\pi/a]$,·Φ_1 and Φ_2 are the force constants for the nearest and second-nearest, respectively. The force constants in 1-dimensional chain are defined as:

$$\Phi(jj') = \frac{d^2V}{dx^2(j)} = -\frac{dF(j)}{dx(j)}$$

Reference

[1] A. Zheludev, S. M. Shapiro, P. Wochner, and L. E. Tanner, Physical Review B **54**, 15045 (1996).

[2] J. Hempelmann, P. C. Müller, P. M. Konze, R. P. Stoffel, S. Steinberg, and R. Dronskowski, Advanced Materials **33** (2021).

[3] S. Lee, K. Esfarjani, T. Luo, J. Zhou, Z. Tian, and G. Chen, Nature Communications **5**, 3525 (2014).
